# Supplementary material for: Variation in high-amplitude events across the human lifespan
Source: Netw Neurosci. 2026 Jan 28;10(1):158–84. doi: 10.1162/NETN.a.515 (PMC12956297; doi:10.1162/NETN.a.515)
Supplement: Supplementary file 1 [file netn-10-1-158-s001.pdf]

Age group	Female (.63%)	Male (.37%)
6 - 15	46 (.47)	52 (.53)
16 - 25	67 (.49)	70 (.51)
26 - 35	23 (.50)	23 (.50)
36 - 45	43 (.73)	16 (.27)
46 - 55	73 (.83)	15 (.17)
56 - 65	62 (.82)	14 (.18)
66 - 75	46 (.67)	23 (.33)

TABLE S1: Age group sex ratio

Communication measures	Description
Euclidean distance (euc)	The Euclidean distance between centers of mass of brain regions.
Path length (pl-bin, pl-wei)	The sum of binarized or weighted edge weights of the shortest path between a source node and a target node.
Communicability (comm-bin, comm-wei)	The weighted sum of walks of all paths between pairs of nodes with weighted denominators (longer walks are more severely penalized).
Cosine similarity (cos-bin, cos-wei)	The similarity of the angle between the two regions' connectivity profiles measured as $S_{xy} = \frac{x \cdot y}{\|x\|\|y\|}$ for the binary and weighted connectivity matrices.
Search information (si-bin, si-wei)	The amount of information (in bits) required to traverse the shortest paths in a network calculated as $S(\pi_{s \to t}) = \log_2[P(\pi_{s \to t})]$.
Path transitivity (pt-wei)	The density of local detours (triangles) that are available along the shortest paths between pairs of nodes.
Mutual information (mi-bin, mi-wei)	The connectivity profile overlap between pairs of nodes calculated as $M_{ij} = \frac{\|\Gamma_i \cap \Gamma_{\lambda_i}\|}{\|\Gamma_i \cup \Gamma_{\lambda_i}\|}$.
Navigability (nav-ms, nav-num)	A communication heuristic with the routing rule of moving towards the node nearest to the target node in metric space (here we use Euclidean distance).
Mean first passage time (mfpt-bin, mfpt-wei)	The number of expected steps in a random walk from node i to arrive at node j.
Flow graph (fg-bin, fg-wei)	The probabilistic flow of random walkers evaluated using a Markov process between nodes i and j at time t using binary and weighted SC matrices.

TABLE S2: Summary description of structural community-derived communication measures

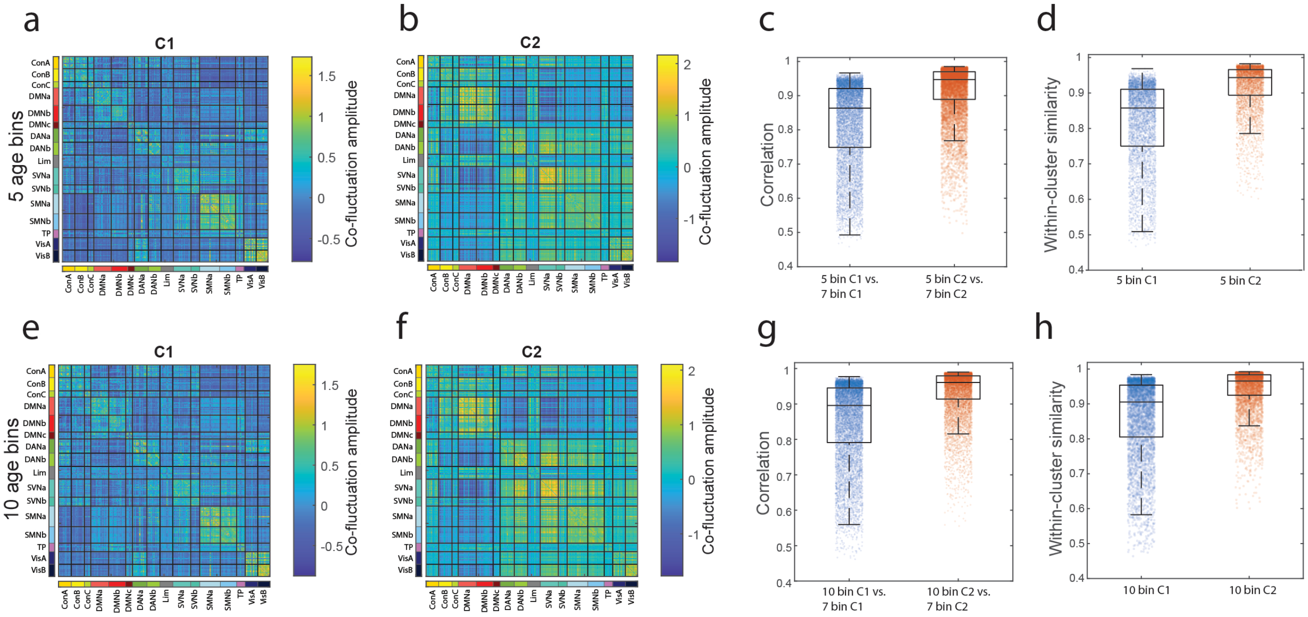

FIG. S1: **Event co-fluctuation patterns using k-means clustering with alternative age bins** Event co-fluctuation patterns across five age bins (age bin = 14 years). (*a*) Mean C1 and (*b*) mean C2 across all age bins. Relative frequency of (*c*) C1 and (*d*) C2 across all five age bins. (*e*) Similarity of C1 and C2 with static FC for five age bins. Event co-fluctuation patterns across ten age bins (age bin = 7 years). (*f*) Mean C1 and (*g*) mean C2 across all ten age bins. Relative frequency of (*h*) C1 and (*i*) C2 across all ten age bins. (*j*) Similarity of C1 and C2 with static FC for ten age bins.

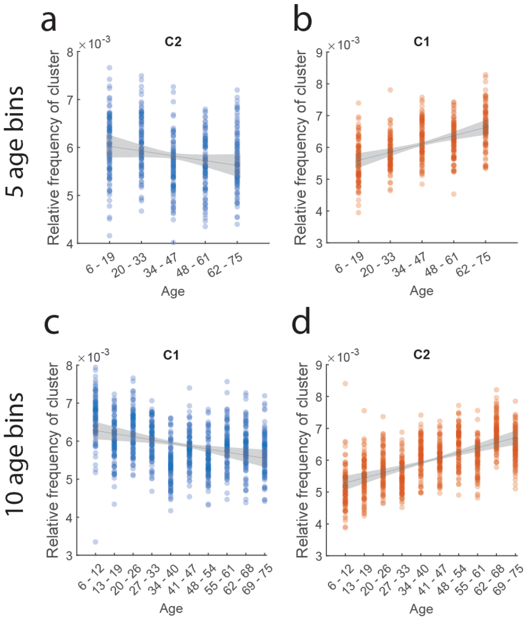

FIG. S2: **Event co-fluctuation pattern frequencies with age in alternative age bins** Relative frequency of (*a*) C1 and (*b*) C2 across all five age bins. Relative frequency of (*c*) C1 and (*d*) C2 across all ten age bins. (*j*) Similarity of C1 and C2 with static FC for ten age bins.

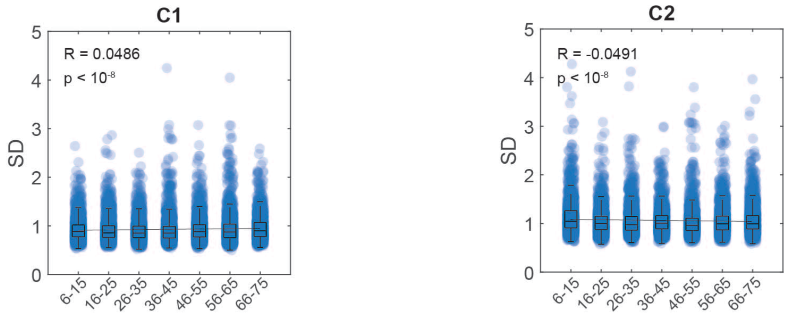

FIG. S3: **Standard deviation of event co-fluctuation patterns within each age bin** (a) Cluster 1 and their standard deviations across subject events within each age bin across 100 iterations. Correlation of standard deviations of cluster 1 with age ($R = 0.0486, p < 10^{-8}$). (b) Cluster 2 and their standard deviations across subject events within each age bin across 100 iterations. Correlation of standard deviations of cluster 2 with age ($R = -0.0491, p < 10^{-8}$).

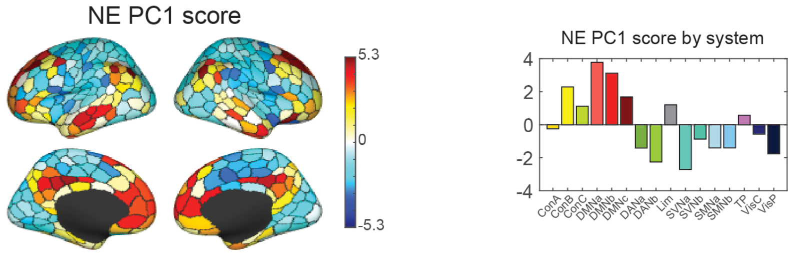

FIG. S4: **Non-event frames and their first principal component** Brain surface plot and the system-level organization of the first principal component score of the mean non-event frames across age groups.

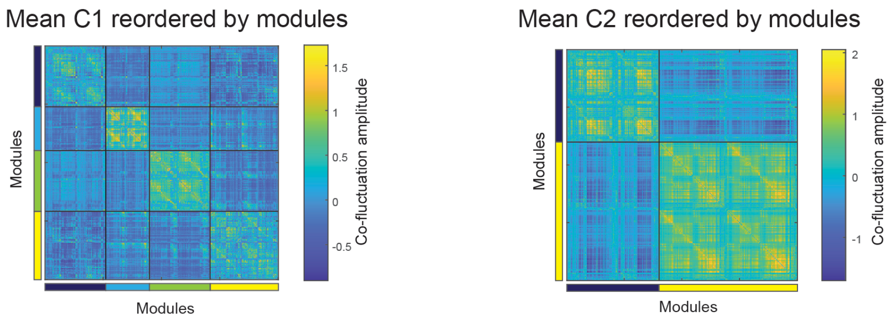

FIG. S5: **Mean event co-fluctuation patterns reordered by modules** The mean C1 and C2 event co-fluctuation patterns across age groups after reordering nodes by their modular labels.

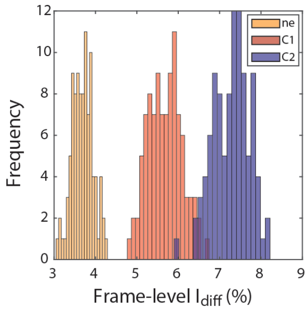

FIG. S6: **Event co-fluctuation patterns and identifiability** *Differential identifiability* of events (C1, C2) and non-events (NE). Frame-level Idiff calculated as the difference between within-subject cluster frame similarity and between subject cluster frame similarity.

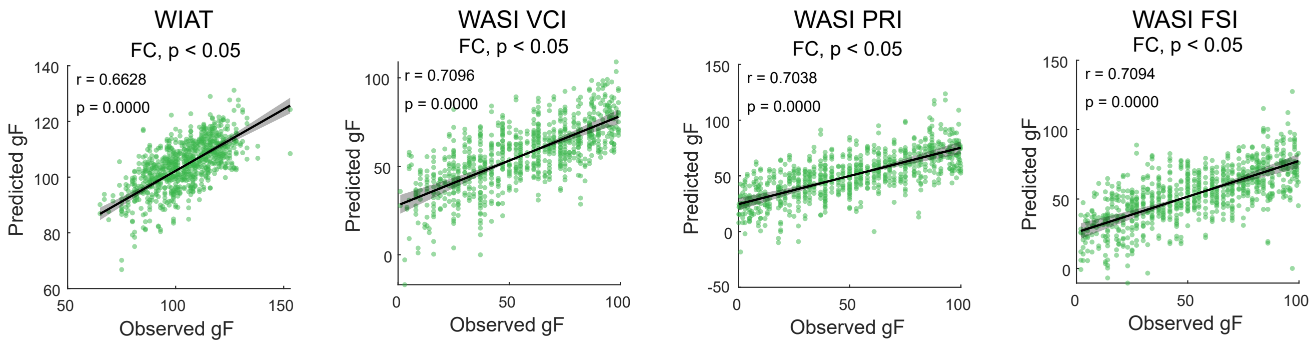

FIG. S7: **Static FC and predictability of achievement and intelligence scores using CPM** Prediction of WIAT, WASI VCI, WASI PRI, WASI FSI scores based on static FC of individuals using connectome-based predictive modeling (CPM).

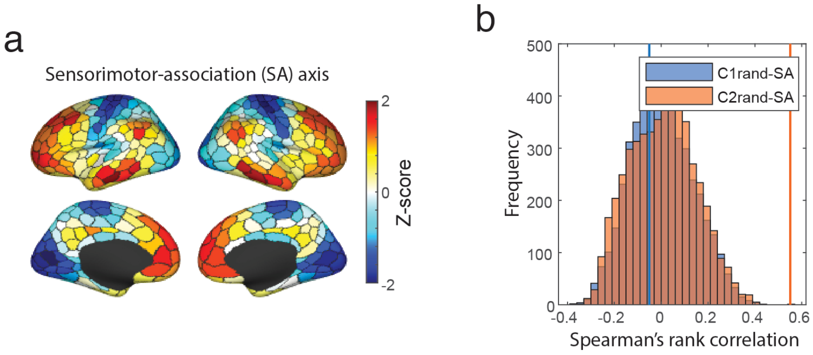

FIG. S8: **Event co-fluctuation patterns and the sensorimotor-association axis** (*a*) Z-score of the whole brain ranking of nodes on the sensorimotor-association (SA) axis. (*b*) Spearman's rank correlation between the average event co-fluctuation patterns' first principal component (PC) and the z-scored global SA axis (C1: orange line; C2: yellow line). Histograms represent the Spearman's rho between average event co-fluctuation patterns' first PCs after spin-test nodal randomization (5000 iterations) and the z-scored global SA axis.

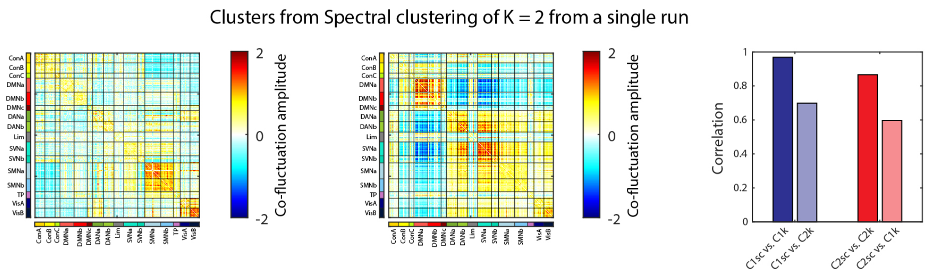

FIG. S9: **Event co-fluctuation patterns from spectral clustering analysis at K = 2, based on a single sampling process** Event co-fluctuation patterns found using spectral clustering analysis at K = 2 from a single sampling process with cluster 1 (left), cluster 2 (middle), and their correlation with clusters found using K-means clustering (right).

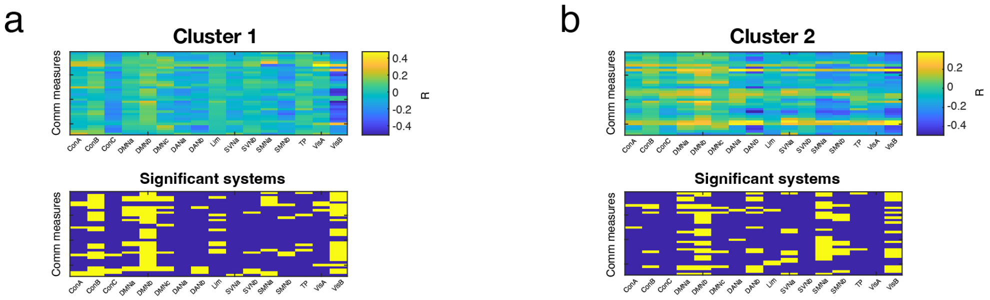

FIG. S10: **Cluster-SC communication measures relationships with age using age group averages** (*a*) Age correlations of communication measures and systems using age group average estimates of cluster 1 and structural connectivity. (*b*) Age correlations of communication measures and systems using age group average estimates of cluster 2 and structural connectivity.

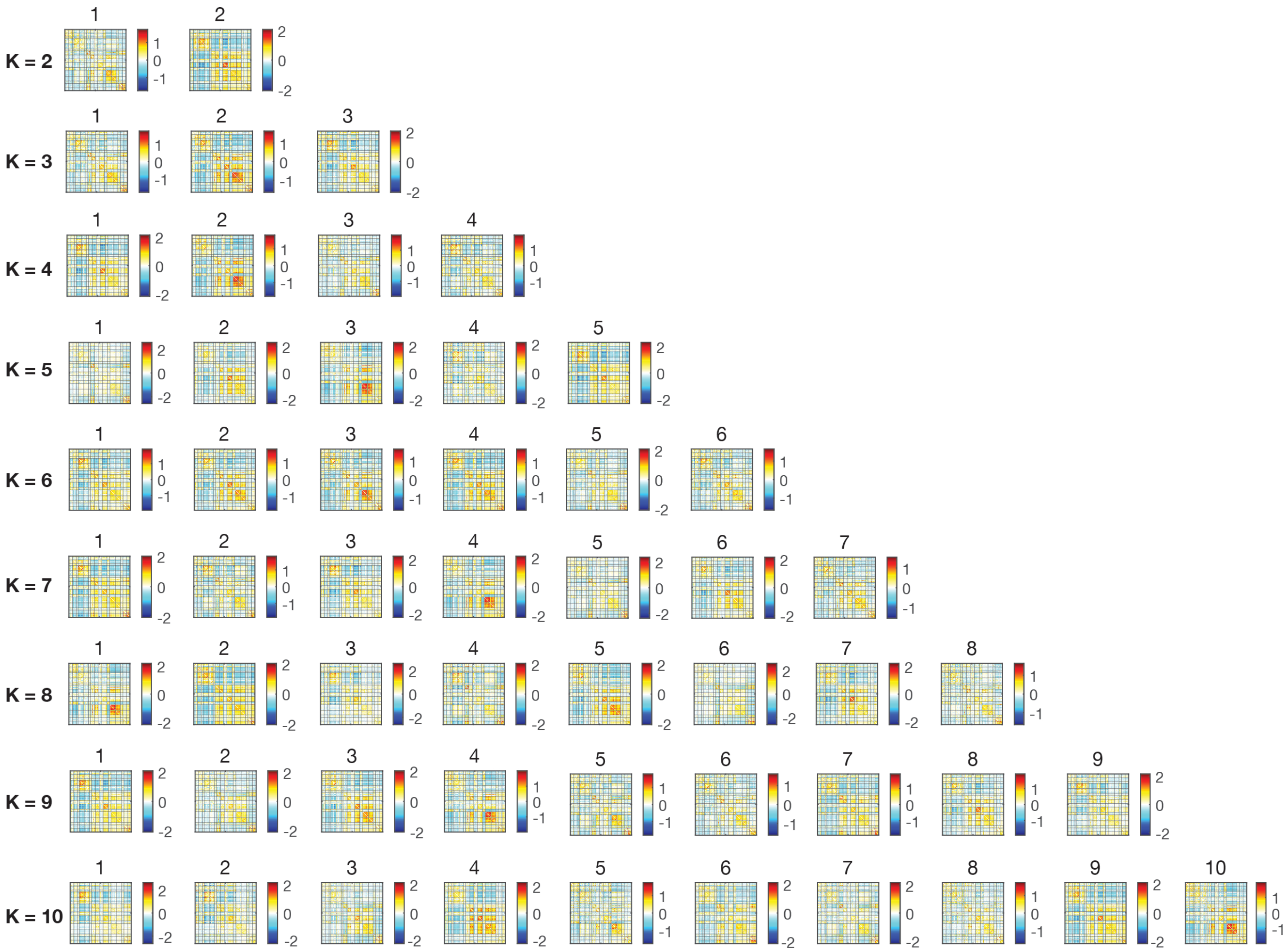

FIG. S11: **Event co-fluctuation patterns after aligning to cluster centroids at** $K = 2 - 10$

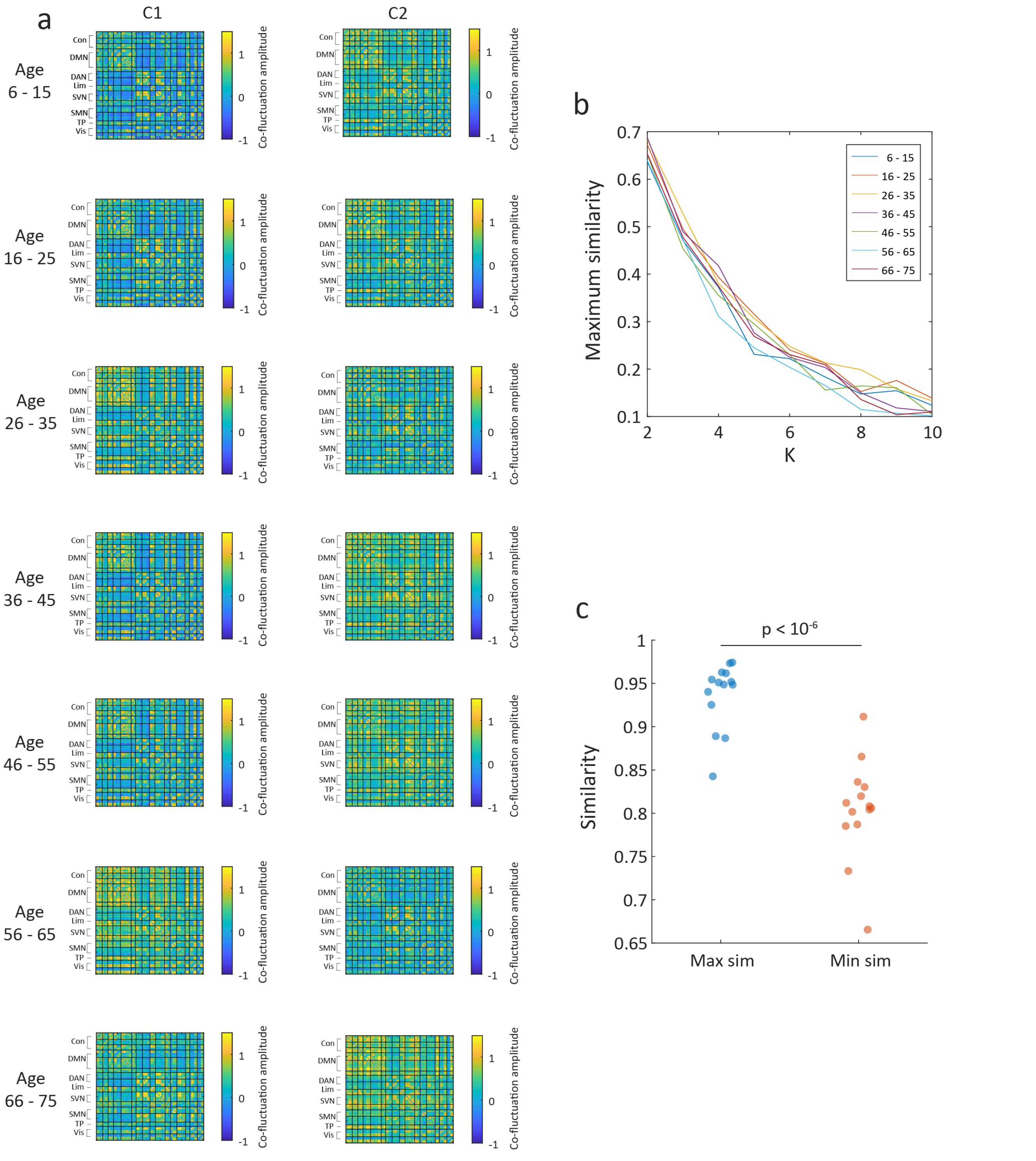

FIG. S12: **K-means clustering for each age group**(*a*) Event patterns of each age group (left: C1, right: C2). (*b*) The maximum similarity across 20 runs of k-means clustering using Pearson correlation (K = 2 − 10). (*c*) Similarity used for aligning clusters across age groups at $K = 2$.

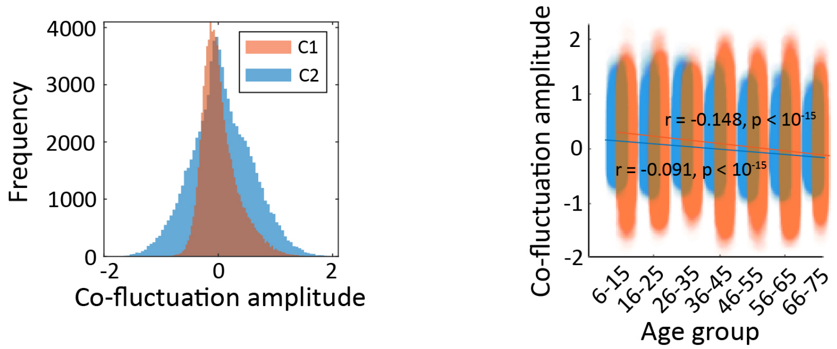

FIG. S13: **Descriptive statistics of event pattern co-fluctuation amplitudes** (*a*) We demonstrate significantly greater co-fluctuation amplitude in C2 than C1 (two-sample t-test, $p < 10^{-15}$). (*b*) Co-fluctuation amplitude in significantly decreased with age in C1 ($r = -0.148, p < 10^{-15}$) and C2 ($r = -0.091, p < 10^{-15}$).

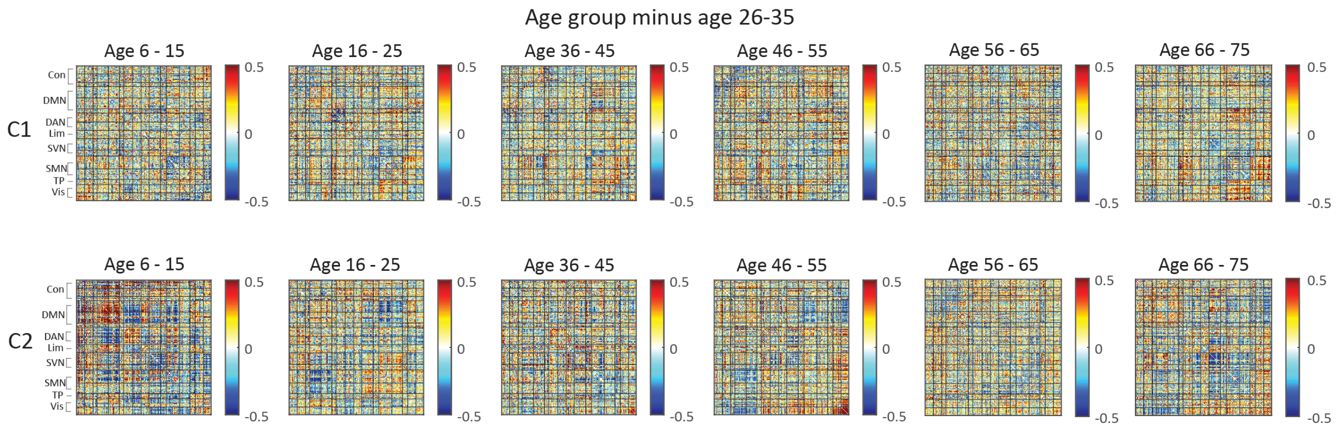

FIG. S14: **Co-fluctuation amplitude difference matrix of each age group minus age group 26-35**

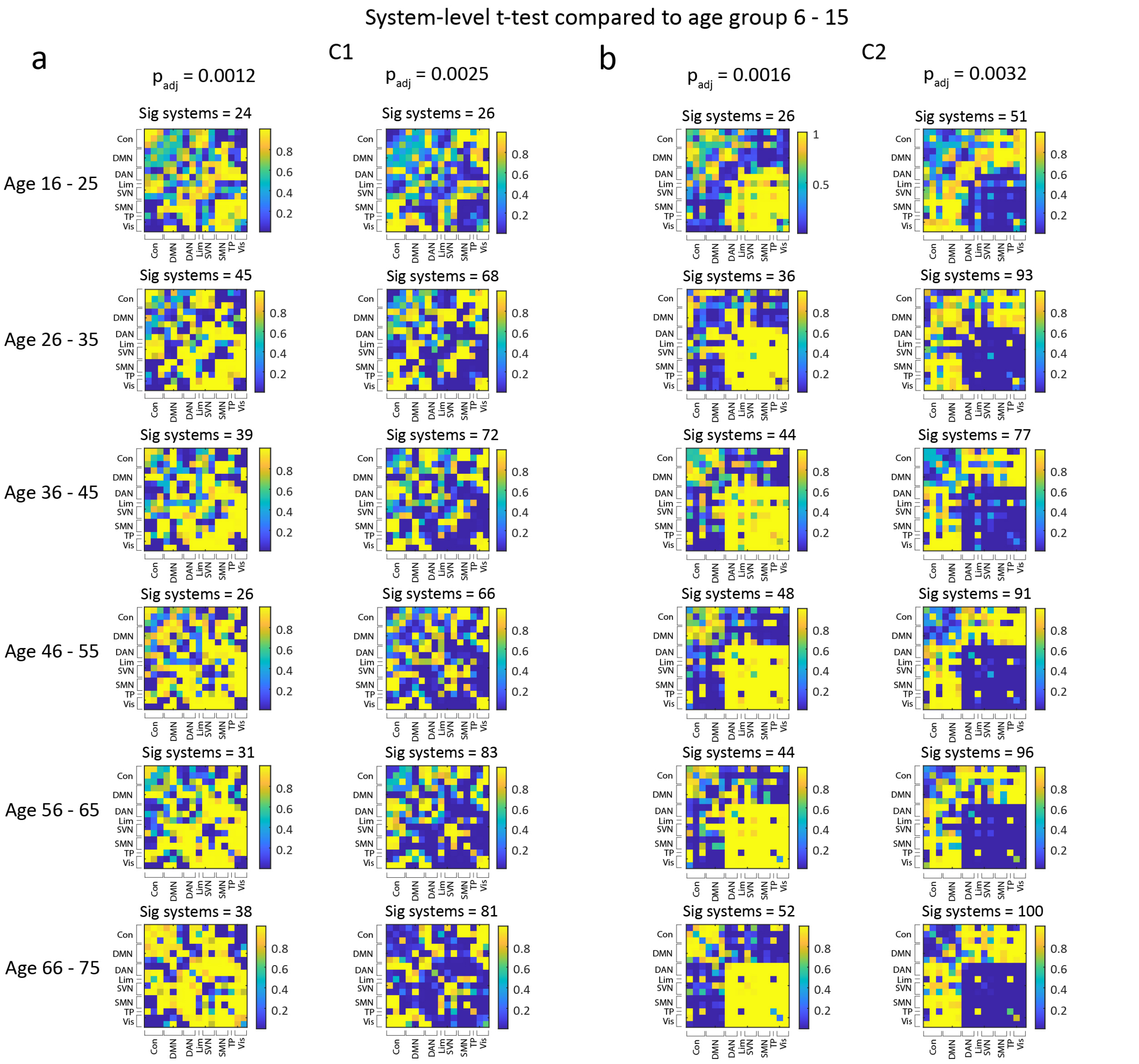

FIG. S15: **System-level two-sample t-tests compared to age group 6 - 15** (*a*) P values of system-level co-fluctuation amplitudes of cluster 1 between age groups with each age group having smaller than (left) and greater than (right) age group 6 - 15. (*b*) P values of system-level co-fluctuation amplitudes of cluster 2 between age groups with each age group having smaller than (left) and greater than (right) age group 6 - 15. The number of significant systems above an FDR-adjusted P value are labeled on top of each subfigure.
